# Non-invasive skin cholesterol testing: a potential proxy for LDL-C and apoB serum measurements

**DOI:** 10.1186/s12944-021-01571-0

**Published:** 2021-10-17

**Authors:** Jiacheng Lai, Yongsheng Han, Chongjian Huang, Bin Li, Jingshu Ni, Meili Dong, Yikun Wang, Qingtong Wang

**Affiliations:** 1 Department of Emergency Medicine, The First Affiliated Hospital of USTC, Division of Life Sciences and Medicine, University of Science and Technology of China, Hefei, China; 2 Emergency and Trauma Center, The International Medical Center, The First Affiliated Hospital, College of Medicine, Zhejiang University, Hangzhou, China; 3 Anhui Provincial Engineering Technology Research Center for Biomedical Optical Instruments, Anhui Institute of Optics and Fine Mechanics, Hefei Institutes of Physical Science, Chinese Academy of Sciences, 230031 Hefei, China; 4 Institute of Clinical Pharmacology, Key Laboratory of Anti-inflammatory and Immune Medicine, Ministry of Education, Collaborative Innovation Center of Anti-inflammatory and Immune Medicine, Anhui Medical University, Hefei, China

**Keywords:** Lipid management, LDL-C, apoB, Skin cholesterol, Low initial LDL-C

## Abstract

**Background:**

Lipid management is the first line of treatment for decreasing the incidence of cardiovascular events in patients with coronary heart disease (CHD), and a variety of indicators are used to evaluate lipid management. This work analyses the differences in LDL-C and apoB for lipid management evaluation, as well as explores the feasibility of skin cholesterol as a marker that can be measured non-invasively for lipid management.

**Methods:**

The prospective study enrolled 121 patients who had been diagnosed with acute coronary syndrome (ACS) at the department of emergency medicine of the First Affiliated Hospital of the USTC from May 2020 to January 2021, and the patients were grouped into Group I (*n*=53) and Group II (*n*=68) according to whether they had comorbid hyperlipidemia and/or diabetes mellitus. All patients were administered 10 mg/day of rosuvastatin and observed for 12 weeks. Lipid management was assessed on the basis of LDL-C and apoB, and linear correlation models were employed to assess the relationship between changes in these well accepted markers to that of changes in skin cholesterol.

**Results:**

Out of 121 patients with ACS, 53 patients (43.80 %) had combined hyperlipidemia and/or diabetes mellitus (Group I), while 68 patients (56.20 %) did not (Group II). Cardiovascular events occur at earlier ages in patients with CHD who are comorbid for hyperlipidemia and/or diabetes (*P*<0.05). LDL-C attainment rate is lower than apoB attainment rate with rosuvastatin therapy (*P*<0.05), which is mainly attributable to patients with low initial LDL-C. Skin cholesterol reduction correlated with LDL-C reduction. (r=0.501, *P*<0.001) and apoB reduction (r=0.538, *P*<0.001). Skin cholesterol reduction continued over all time points measured.

**Conclusions:**

Examination of changes in apoB levels give patients with low initial LDL-C more informative data on lipid management than LDL-C readings. In addition, non-invasive skin cholesterol measurements may have the potential to be used independently for lipid management evaluation.

## Introduction

Over the past decades, China has achieved remarkable socio-economic development. However, in terms of health, it has shown a rapid increase in the morbidity and mortality of chronic diseases that are mainly part of atherosclerotic cardiovascular disease (ASCVD) [1]. Dyslipidemia is a major contributor to the incidence of cardiovascular events in ASCVD, and the incidence of cardiovascular events in patients with coronary heart disease (CHD) is significantly reduced after receiving lipid-lowering therapy [2, 3]. Numerous associations have published varying lipid guidelines to aid in cardiovascular event risk management. What all the guidelines agree upon is that low-density lipoprotein-cholesterol (LDL-C), the most common clinical marker, is able to evaluate the lipid management of the vast percentage of patients. Yet, in some cases (such as when a patient has a comorbidity of hypertriglyceridemia), the commonly-used Friedewald equation gives an erroneous diagnosis [4]. The 2019 European Society of Cardiology (ESC) lipid guidelines already recommend using apolipoprotein B (apoB) as an evaluation marker in obese people or in the case of combined hyperlipidemia and/or diabetes [5]. Recently, a series of studies has proposed some new equations and assays (such as use of direct LDL-C measurement, nuclear magnetic resonance spectrometry, the Martin equation, and the Sampson equation) for accurately calculating serum LDL-C levels in patients with combined hyperlipidemia and proposed the use of small dense low-density lipoprotein-cholesterol (sdLDL-C) as an alternative to LDL-C for cardiovascular event risk assessment [6, 7]. Despite the complementarity of these evaluation markers, there is still a lack of a single marker that can provide a comprehensive assessment of all patients with CHD.

In 2005, Tzou’s team introduced skin cholesterol to assess the risk of cardiovascular events [8]. Studies have shown a strong connection between skin cholesterol levels and serum cholesterol, and clinical study has shown cholesterol accumulation in the skin is associated with risk of atherosclerotic disease. This suggests that skin cholesterol has a theoretical basis for cardiovascular event risk assessment [8–13]. In this study a novel, rapid and non-invasive skin cholesterol detection system that utilizes fluorescence spectroscopy was employed to quickly detect the skin cholesterol level without the need for biopsy [14, 15]. The aim of this study was to investigate the disparities of LDL-C and apoB in the evaluation of lipid management in patients with CHD and study the potential value of skin cholesterol detection in lipid management.

## Methods

### Study population

**Fig. 1:**
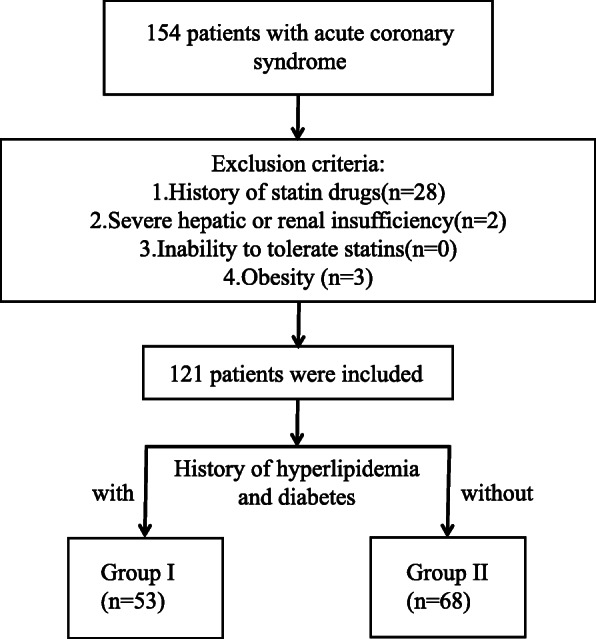
Patients’ selection process: Firstly, a total of 154 patients with ACS were initially enrolled; secondly, 121 patients were selected in the study after screening; finally, these patients were divided into two groups

154 patients diagnosed with acute coronary syndrome (ACS) between January 2020 and April 2021 were included in the study. According to the exclusion criteria, 121 patients were finally enrolled (Fig. 1). Consenting patients who were diagnosed and treated with a primary cardiovascular event at the First Affiliated Hospital of the USTC, in accordance with the criteria of ACS diagnosis and treatment guidelines [16, 17] were enrolled in the study. Informed consent was obtained and signed. Patients with a history of statin drugs, inability to tolerate statins, severe hepatic or renal insufficiency, and/or obesity (BMI>28 kg/m^2^) were excluded from the study.

Patients were divided into two groups based on the 2019 ESC lipid guidelines which recommend that patients with hyperlipidemia or diabetes have apoB evaluated due to false positive results with LDL-C readings [5]. Group I (*n*=53): patients had comorbid diabetes and/or hyperlipidemia; and Group II (*n*=68): patients did not have diabetes or hyperlipidemia.

All patients received 10 mg/day of rosuvastatin as the starting lipid-lowering regimen. Patients with LDL-C <1.8 mmol/L and ≥50 % reduction at the 4th week of follow-up were considered to have achieved the LDL-C standard [18]. ApoB attainment was defined as apoB <65 mg/dl by the 4th week of follow-up [5]. According to the Chinese lipid guidelines, the 12th week was recommended as the follow-up endpoint [1].

### Non-invasive skin cholesterol detection

**Fig. 2:**
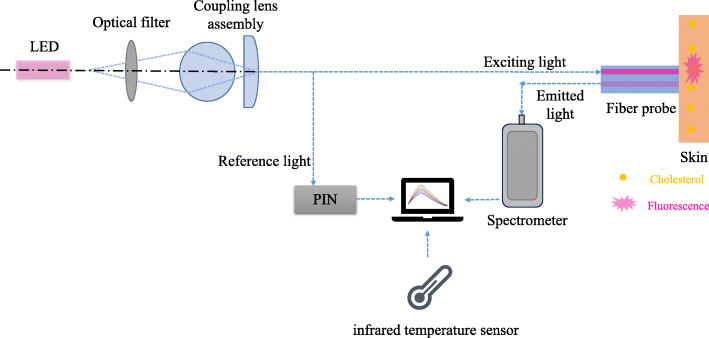
The principle of non-invasive skin cholesterol testing system

The protocol was approved by Ethics Committee of University of Science and Technology of China (Registration No. 1804h08020291(2018-05-30)). Quantification of skin cholesterol was determined by specifically binding reagents to the skin, causing the cholesterol to fluoresce under excitation light (Fig. 2) [14].

**Fig. 3:**
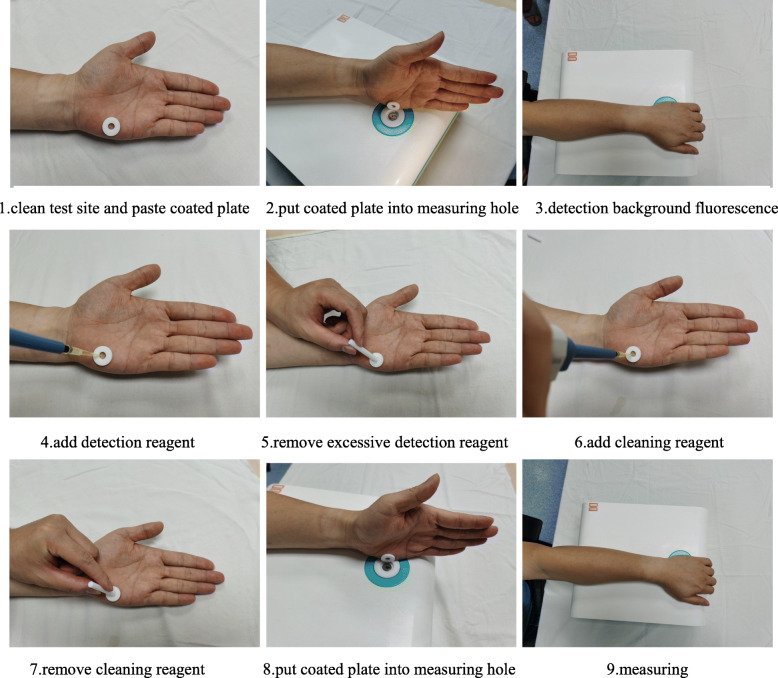
Detection process of non-invasive skin cholesterol testing system

The hypothenar area of the left hand was selected for detection because the skin of the left palm is less abrasive and has fewer melanocytes than the rest of the body, allowing for the detection results to be more stable [15]. Testing was performed by first cleaning the test site with an alcohol swab, applying a plastic-coated annulus to the test site on the skin surface, and placing the examined part on the measuring hole of the detection system to measure the skin background spectrum. Once the background signal was determined, the detection reagent was added drop by drop to fill the annulus. After 60 s, excessive detection reagent was removed with a sterile cotton swab, and the cleaning reagent was added to the annulus for 30 s before removal with a sterile cotton swab. The treated portion of skin was then placed over the measuring hole of the detection system. A comparison of the two spectra allows for a quantification of skin cholesterol [14] (Fig. 3).

### Data collection

Serum lipids were also collected and analyzed [2]. The reduction of skin cholesterol, LDL-C, and apoB were calculated from subtracting the follow-up values from the initial values. Four follow-up nodes were established for this study: the 2nd week of statin therapy, the 4th week of statin therapy, the 8th week of statin therapy, the 12th week of statin therapy.

### Statistical analysis

Repeated measure 1-way ANOVAs with Tukey post-tests were performed to compare data from different time points. For the categorical variables, the comparison was made using the chi-square test (χ^2^ test) / Fisher’s exact test. Paired t-test was used for comparison of continuous variables within the group. Linear correlation analysis was performed between skin cholesterol reduction and LDL-C reduction, and skin cholesterol reduction with apoB reduction. A *p-*value <0.05 was rated as statistically significant.

## Results

### Characteristics of coronary heart disease patients with vs. without comorbidities of diabetes and/or hyperlipidemia

The study enrolled 121 patients with ASCVD. Group I contained 13 patients with hyperlipidemia, 7 patients with diabetes, 3 patients with hyperlipidemia and diabetes, 6 patients with hypertension and hyperlipidemia, 18 patients with hypertension and diabetes, and 6 patients with hypertension, hyperlipidemia and diabetes.

The mean age of patients in Group I was 56.43 years old, which is significantly lower than the mean age of 62.26 years old in the Group II (*P*<0.05). There were 24 smokers in Group I and 22 in Group II, all of whom were male. Smokers accounted for 45.28 % and 39.29 % of male patients in each group, respectively (*P*<0.05). No statistical significance was found regarding gender composition nor history of hypertension (Table 1).

**Table 1:** General patient information

	Group I (n=53)	Group II (n=68)	t/χ^2^	*P*
Age (years old)	56.43±13.23	62.26±13.00	2.429	0.017
Male (n/%)	40(75.47 %)	56(82.35 %)	0.860	0.241
Male smokers (n/%)	24(45.28 %)	22(39.29 %)	4.012	0.036
History of hypertension (n/%)	30(56.60 %)	34(50.00 %)	0.521	0.295
TG (mmol/L)	2.25±2.04	1.44±0.51	3.991	<0.001
HDL-C (mmol/L)	0.88±0.20	0.91±0.26	-0.745	0.458
LDL-C (mmol/L)	3.05±1.79	2.64±0.87	1.630	0.106
number of low initial LDL-C cases(n/%)	9(16.98 %)	12(17.65 %)	0.009	0.560
apoA-1 (g/L)	1.28±0.20	1.26±0.22	0.563	0.574
apoB (g/L)	1.01±0.48	0.86±0.23	2.329	0.022
Lp(a) (g/L)	344.24±297.91	260.63±207.07	1.701	0.093
Initial skin cholesterol	0.32±0.08	0.31±0.08	0.538	0.592

Low initial LDL-C: LDL-C < 1.8mmol/L at admission

Patients in Group I had higher triglycerides (TG) (2.25±2.04 mmol/L ) than Group II (1.44±0.51 mmol/L) at admission (*P*<0.001). The apoB on admission was higher in Group I (1.01±0.48 g/L) than in the Group II (0.86±0.23 g/L) (*P*<0.05). There were not any differences between groups in high density lipoprotein cholesterol (HDL-C), LDL-C, number of low initial LDL-C cases, apolipoprotein A-1 (apoA-1), lipoprotein (a) (Lp (a)), nor initial skin cholesterol at admission (Table 1).

### Comparison of LDL-C and apoB in the evaluation of lipid management results between two groups

The 121 patients enrolled were evaluated for lipid management based on whether the desired goals for LDL-C and apoB were achieved at the 4th week of follow-up. The 53 patients in Group I contained 30 patients with apoB (+) and 23 patients with apoB (-), 20 patients with LDL-C(+) and 33 patients with LDL-C (-) (*P*<0.05). The 68 patients from Group II included 33 patients with apoB (+) and 35 patients with apoB (-), 20 patients with LDL-C (+) and 48 patients with LDL-C (-) ( *P*<0.05) (Table 2).

**Table 2:** Differences in LDL-C and apoB evaluation results

	Group I			Group II		
	LDL-C (+) (n)	LDL-C (-) (n)	Total (n)	LDL-C (+) (n)	LDL-C (-) (n)	Total (n)
apoB (+)	20	10	30	18	15	33
apoB (-)	0	23	23	2	33	35
Total	20	33	53	20	48	68
*P*	0.04			0.017		

LDL-C (+): patients met criteria by LDL-C assessment; LDL-C (-): patient does not meet criteria by LDL-C assessment

apoB (+): patients met criteria by apoB assessment; apoB (-): patient does not meet criteria by apoB assessment

Analysis of the 9 patients in Group I with low initial LDL-C revealed that there were 0 patients with LDL-C (+) /apoB (+), 0 patients with LDL-C (+) /apoB (-), 6 patients with LDL-C (-) /apoB (+), and 3 patients with LDL-C (-)/apoB (-) (*P*<0.05). The 12 patients from Group II included 1 patient with LDL-C (+)/apoB (+), 0 patient with LDL-C (+)/apoB (-), 6 patients with LDL-C (-)/apoB (+), 5 patients with LDL-C (-)/apoB (-) (*P*<0.05) (Table 3).

**Table 3:** Differences in the evaluation results of LDL-C and apoB in two groups of patients with low initial LDL-C

	Group I			Group II		
	LDL-C (+) (n)	LDL-C (-) (n)	Total (n)	LDL-C (+) (n)	LDL-C (-) (n)	Total (n)
apoB (+)	0	6	6	1	6	7
apoB (-)	0	3	3	0	5	5
Total	0	9	9	1	11	12
*P*	0.005			0.014		

### Association of skin cholesterol with LDL-C, apoB and follow-up time

**Fig. 4:**
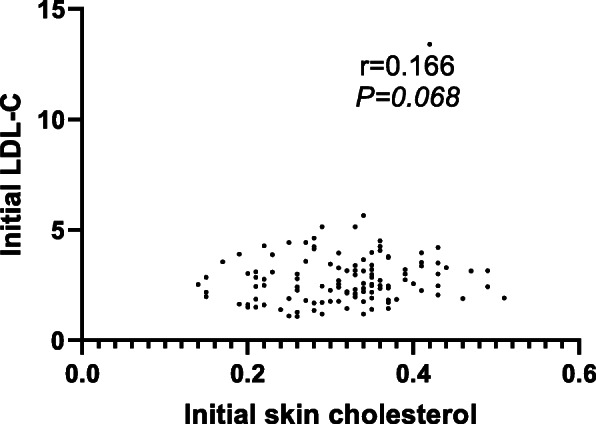
Linear correlation analysis of association between initial skin cholesterol and initial LDL-C (Group I and Group II)

**Fig. 5:**
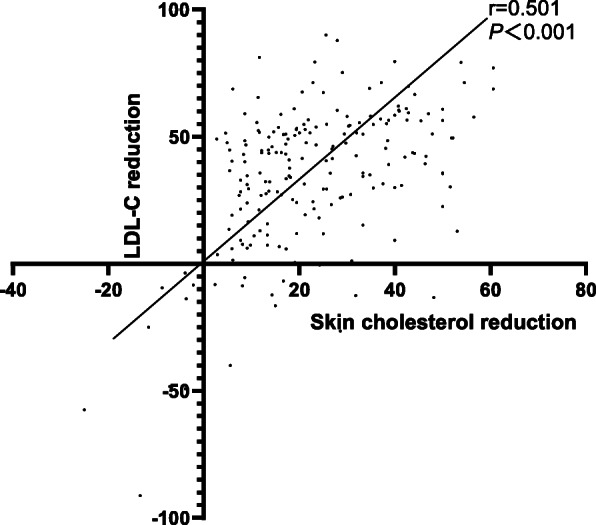
Linear correlation analysis of association between skin cholesterol reduction and LDL-C reduction (Group I and Group II)

**Fig. 6:**
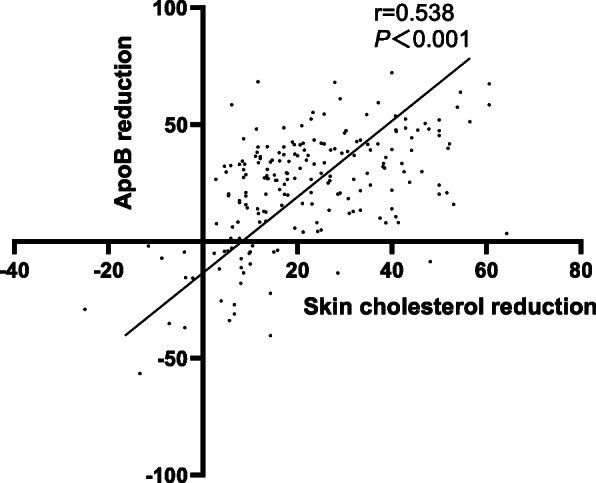
Linear correlation analysis of association between skin cholesterol reduction and initial apoB reduction (Group I and Group II)

Although the linear correlation analysis showed lack of correlation between initial skin cholesterol and initial LDL-C (Fig. 4), the correlation between skin cholesterol reduction and LDL-C reduction was significant (r=0.501, *P*<0.001) implying that skin cholesterol reduces in conjunction with LDL-C reduction (Fig. 5). A similar linear correlation was seen between skin cholesterol reduction and apoB reduction (r=0.538, *P*<0.0001) (Fig. 6).

**Fig. 7:**
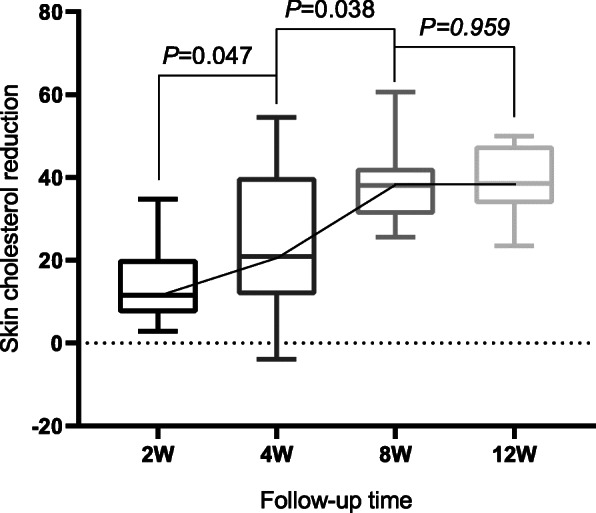
1-way ANOVAs of association between skin cholesterol reduction and follow-up time in Group I apoB (+) patients; no significant correlation between kin cholesterol reduction and follow-up time in Group I apoB (-) patients occured

**Fig. 8:**
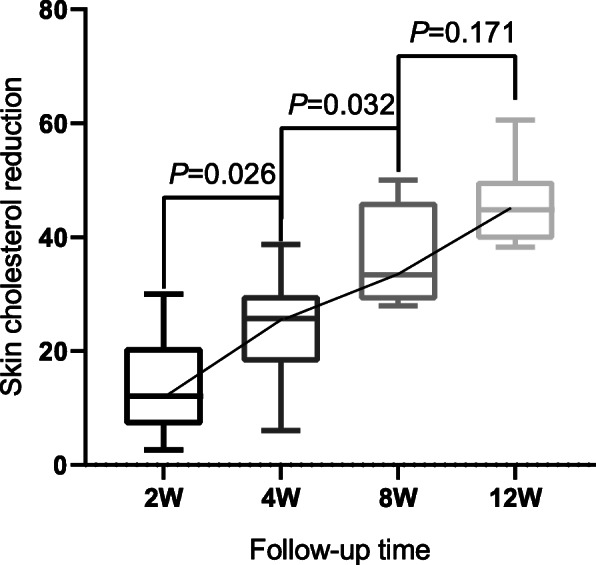
1-way ANOVAs of association between skin cholesterol reduction and follow-up time in Group II LDL-C (+) patients; No significant correlation between kin cholesterol reduction and follow-up time in Group II LDL-C (-) patients occured

ApoB (+) patients in Group I had a continual reduction in skin cholesterol over the first 8 weeks (*P*=0.047, *P*=0.038), and portrayed a stable trend between the 8th week and 12th week (Fig. 7). Furthermore, skin cholesterol in Group II LDL-C (+) patients (*P*=0.026, *P*=0.032, *P*=0.171) decreased over all time points measured (Fig. 8).

## Discussion

With the progress of society, morbidity and mortality from CHD are increasing. Dyslipidemia is closely related to the development of CHD. Currently, guidelines recommend LDL-C as a therapeutic marker for the evaluation of lipid management and cardiovascular event risk status [5, 20]. However, the single marker use of LDL-C calculated by the Friedewald equation is inadequate in specific conditions that are prone to false positives. Although Sampson et al. has proposed a calibration formula to more accurately calculating serum LDL-C levels in hyperlipidemic patients [21], the European society of cardiology has proposed the introduction of apoB to assist in the evaluation [5]. ApoB and LDL-C are similar in clinical significance, with apoB being preferred to LDL-C in the assessment of ASCVD risk with diabetic comorbidity [22–25]. In recent years, with the increasing number of patients with CHD who have comorbidities of hyperlipidemia and/or diabetes mellitus [26–28], the importance of apoB has been elevated. Further understanding of the differences in the effects of marker evaluation and the exploration of single markers would be beneficial for the management of lipids in patients with CHD in clinical applications.

In this study, patients with comorbid hyperlipidemia and/or diabetes accounted for 43.80 % of the patients recruited during the same period, and the mean age of onset was 56.43 years old in Group I, significantly lower than the average 62.26 years old in Group II. This suggested that patients with more risk factors suffered earlier CHD incidents than those with fewer risk factors, similar to the findings of recent studies [29].

The assessment of lipid management outcomes using LDL-C and apoB showed significant differences between Group I and Group II. Further analysis revealed differences mainly in the results of the evaluation of patients with low initial LDL-C. Patients with low initial LDL-C on a 10 mg/day rosuvastatin regimen had trouble achieving the desired goal after 4 weeks of treatment and had difficulty meeting the LDL-C evaluation compliance requirements. However, those patients had absolute serum LDL-C values within the ideal range and were at low risk of recurrent cardiovascular events. This data suggests that it might be more feasible to use apoB to assess lipid management status in patients with initial low LDL-C.

The study here within evaluated skin cholesterol as another marker. In a previous study, the team analyzed 342 study subjects (110 atherosclerosis patients, 117 high-risk and 115 low-risk individuals) and found that skin cholesterol was associated with atherosclerotic disease [15]. Skin cholesterol was associated with a variety of factors, such as age [30], gender [31], genetic disorders [32, 33], and degree of skin tanning [34]. The lack of correlation between initial skin cholesterol and initial LDL-C in the study might be due to these confounding multiple factors in the tested population. Therefore, the effect of individual differences was normalized by calculating the skin cholesterol reduction. By doing so, reductions in both traditional serum markers and a potential non-invasive marker for lipid management could be significantly correlated to each other. According to the guideline recommendation, apoB grouping was used for patients in Group I, and it was found that the rate of skin cholesterol reduction increased over time in patients who met the standard, while the rate of skin cholesterol reduction in patients who did not meet the standard was poorly associated with the follow-up time. Patients in Group II were grouped using LDL-C, and the trend in skin cholesterol reduction with time was similar to that of Group I. This reflects the ability of skin cholesterol to reveal whether LDL-C and apoB standards are met, which in combination with clinical findings suggests the potential value of skin cholesterol for lipid management evaluation in patients with CHD.

As research progresses, new LDL-C calculation equations and assays have been proposed [6, 7, 21]. The Martin equation was a further development of the Friedewald equation that reduced the calculation error for low LDL-C levels. In addition, direct LDL-C assays can compensate for the large detection error of the Friedewald equation when TG >400 mg/dL. Moreover, the Sampson equation is a more comprehensive equation that studies have shown can be used to calculate LDL-C in patients with any TG level. Nuclear magnetic resonance spectrometry can detect both LDL-C concentration and LDL-C particle size which is a good guide for further response to ASCVD risk. Compared with these equations and assays, skin cholesterol has its own unique advantages: it eliminates the need for precise measurement of serum LDL-C while determining the effect of statin therapy; it can be applied to all patients, including those with low initial LDL-C levels; it is a simple, non-invasive test that takes only a few minutes; and the test instrument is portable and ideal for use by family physicians.

## Study strengths and limitations

Evidence is provided within the current work which supports that apoB can be used for lipid management in patients with low initial LDL-C. Data is also provided depicting that skin cholesterol reduction is positively correlated with LDL-C reduction and apoB reduction. Therefore, skin cholesterol has potential value for lipid management evaluation in patients with CHD. Moreover, since the basic information of the patients in the study is similar to other studies, this work can be expanded to further develop non-invasive skin cholesterol testing in the future [35, 36].

This study was limited by the follow-up time of each patient. This is due to guidelines requesting that lipid-lowering regimens need to be adjusted for patients who remain poorly controlled on lipid-lowering medications for 12 weeks. In addition, the study suffered from the shortcomings of a single center, small sample, and short duration.

## Conclusions

The current study analyzed the differences in the application of LDL-C and apoB and provided guidance for clinical work. In addition, the study identified the potential value of skin cholesterol for lipid management in patients with ACS, providing a reference for the selection of markers for lipid management. Moreover, use of skin cholesterol as a marker could enhance lipid management in patients with ACS since the method of detection is non-invasive.

## Data Availability

Data and materials are available online or from corresponding authors upon request.

## Abbreviations

ASCVD: atherosclerotic cardiovascular disease
CHD: coronary heart disease
**LDL-C**: low-density lipoprotein-cholesterol
ESC: European Society of Cardiology
apoB: apolipoprotein B
ACS: acute coronary syndrome
HDL-C: high density liptein cholesterol
apoA-1: apolipoprotein A-1
Lp (a): lipoprotein (a)
**low initial LDL-C**: LDL-C<1.8mmol/L at admission
**LDL-C (+)**: patients met criteria by LDL-C assessment
LDL-C (-): patient does not meet criteria by LDL-C assessment
apoB (+): patients met criteria by apoB assessment
apoB (-): patient does not meet criteria by apoB assessment
